# Synergistic Malaria Parasite Killing by Two Types of Plasmodial Surface Anion Channel Inhibitors

**DOI:** 10.1371/journal.pone.0149214

**Published:** 2016-02-11

**Authors:** Margaret Pain, Alexandra W. Fuller, Katherine Basore, Ajay D. Pillai, Tsione Solomon, Abdullah A. B. Bokhari, Sanjay A. Desai

**Affiliations:** The Laboratory of Malaria and Vector Research, National Institute of Allergy and Infectious Diseases, National Institutes of Health, Rockville, Maryland, United States of America; Institut national de la santé et de la recherche médicale - Institut Cochin, FRANCE

## Abstract

Malaria parasites increase their host erythrocyte’s permeability to a broad range of ions and organic solutes. The plasmodial surface anion channel (PSAC) mediates this uptake and is an established drug target. Development of therapies targeting this channel is limited by several problems including interactions between known inhibitors and permeating solutes that lead to incomplete channel block. Here, we designed and executed a high-throughput screen to identify a novel class of PSAC inhibitors that overcome this solute-inhibitor interaction. These new inhibitors differ from existing blockers and have distinct effects on channel-mediated transport, supporting a model of two separate routes for solute permeation though PSAC. Combinations of inhibitors specific for the two routes had strong synergistic action against *in vitro* parasite propagation, whereas combinations acting on a single route produced only additive effects. The magnitude of synergism depended on external nutrient concentrations, consistent with an essential role of the channel in parasite nutrient acquisition. The identified inhibitors will enable a better understanding of the channel’s structure-function and may be starting points for novel combination therapies that produce synergistic parasite killing.

## Introduction

Malaria remains a leading cause of morbidity and mortality worldwide. Because an effective vaccine against malaria is not available, prophylaxis and treatment rely on antimalarial drugs. With evolving parasite resistance to most drugs despite use of combination therapies [1], there are not enough treatment options to achieve sustained global malaria control. Thus, critical parasite activities that have not yet been targeted should be characterized and evaluated for therapeutic potential.

The plasmodial surface anion channel (PSAC) is an attractive target as it mediates uptake of required nutrients at the host membrane and is a prime example of how the virulent *Plasmodium falciparum* parasite remodels its host cell, the human erythrocyte [2–4]. While early studies reported upregulated human transporters [5–8], molecular studies with transport mutants and specific inhibitors have more recently established that PSAC is responsible for the uptake of most solutes [9,10]. Recently, the parasite *clag* gene family has been identified as a determinant of PSAC activity through genetic mapping with isolate-specific inhibitors as well as gene silencing and site-directed mutagenesis experiments [11–15]. The encoded protein is integral to the host erythrocyte membrane, suggesting direct involvement in channel formation and a role in transport. Nevertheless, the full composition and structure of functional channels at the host membrane remains unclear.

Channel-mediated increases in erythrocyte permeability may be one of the most essential exported parasite activities [16]. Most convincingly, both increased permeability after infection and the associated *clag* genes are strictly conserved in all plasmodial species studied to date [17,18]; both are absent from other apicomplexan parasites, implicating an adaptation unique to *Plasmodium* spp. [19]. The presence of multiple *clag* paralogs in each species as well as epigenetic regulation of family members also suggest an essential role [13,14,20,21], as pathogens use these strategies to evade host immune responses and protect critical activities [22,23]. Finally, both specific and nonspecific inhibitors of this channel produce *in vitro* parasite killing at concentrations that prevent nutrient uptake [2,10].

Nevertheless, development of therapies targeting PSAC is limited by a still rudimentary understanding of inhibitor action and the effects of nutrient restriction on parasite development. One unexpected finding is that known PSAC inhibitors have differing efficacies against a conserved subset of solutes [24]. A consistent pattern of reduced inhibitor efficacy and restored activity in uptake measurements using solute mixtures excludes flux through multiple unrelated channels; instead, these findings suggested two distinct mechanisms of solute transport through a single channel. Examination of solute structures or physicochemical properties have not revealed how or why PSAC may distinguish between the two groups of permeant solutes [25].

Here, we examined possible mechanisms and physiological relevance of these solute-inhibitor interactions by executing a high-throughput screen for a novel class of PSAC inhibitors. The identified hits are chemically distinct from existing inhibitors and have distinct effects on solute transport. Importantly, combinations of PSAC inhibitors from the two classes exhibit potent synergistic activity against *in vitro* parasite growth. These findings define two mechanisms of transport through PSAC, reveal that both transport components are used by developing intracellular parasites, and should guide rational drug development aimed at targeting nutrient acquisition.

## Materials and Methods

### Parasite cultures

*P*. *falciparum* laboratory lines were cultivated by standard methods using O^+^ human erythrocytes (Interstate Blood Bank, Memphis, TN) and RPMI 1640 medium (Gibco, Waltham, MA) supplemented with 50 mg/L hypoxanthine and either 10% pooled human serum or 0.5% NZ microbiological BSA (MP Biomedicals, Santa Ana, CA). Cultures were maintained under 5% O_2_, 5% CO_2_, 90% N2 at 37°C. The Indo strain was used for high-throughput screening, but secondary studies using the Dd2, HB3, and FCB lines yielded similar results, which were combined for statistical analyses.

### Transport assays

Transport measurements used synchronous trophozoite-stage infected erythrocytes enriched from *P*. *falciparum* cultures by Percoll/sorbitol separation to > 95% parasitemia. Enriched cells were washed in HBS (150 mM NaCl, 20 mM Na-HEPES, 0.1 mg/mL BSA, pH 7.5) and used for continuous tracking of osmotic lysis in permeant solutes as described [10]. PSAC-mediated uptake was initiated by resuspension of cells in osmotic lysis solutions containing 20 mM Na-HEPES, 0.1 mg/mL BSA with permeant solute or salt added at 280–290 mOsm; where present, inhibitors were added from concentrated stocks in DMSO. Osmotic swelling and lysis at 37°C was then kinetically tracked by recording the transmittance of 700 nm light through the 1 mL cell suspension (Beckman Coulter spectrophotometer, Indianapolis, IN). Regular manual resuspension was used to minimize the effects of cell settling. Solute permeability was calculated using locally developed script as described [26]. The data are presented with normalization to inhibitor-free controls included in each experiment.

Transport studies were also performed with *P*. *knowlesi* (H strain) after *in vitro* cultivation with erythrocytes collected from healthy rhesus monkeys (*Macaca mulatta*) in compliance with National Institutes of Health guidelines under an Animal Care and Use Committee-approved protocol [18]. These parasites were identically cultivated except that the culture medium was supplemented with 1.0% NZ microbiological BSA. Trophozoite-stage infected cells were harvested, enriched, and used for osmotic lysis kinetic recordings as above.

### High-throughput screening

A cell-based screen for residual transport inhibitors was performed using the above transmittance assay in 384-microplate format. Enriched infected cells were resuspended in HBS at 1% hematocrit prior to dispensing 20 uL/well into microplates (Matrix Wellmate, Thermo Fisher Scientific, Waltham, MA). Screening compounds were dispensed from mother plates containing 10 mM DMSO stocks using a 100 nL pin transfer. After a ≥ 20 min incubation, 80 uL of prewarmed lysis solution containing 145 mM PhTMA-Cl, 20 mM Na-HEPES, 0.1 mg/mL BSA, 200 μM furosemide, pH 7.5 was dispensed to each well. Plates were vortexed and incubated at 37°C for 3 h prior to absorbance measurements (700 nm, Synergy HT, Biotek, Winooski, VT); readings were also taken at 1 h and 17 h to confirm kinetics. DMSO-only negative control wells were included in each plate. Positive control plates, containing cells in HBS with 1.6 mM furosemide, were included on each screening day. Activities were calculated according to % block of residual PhTMA transport = 100*(*A*_*cpd*_*− Ā*_*neg*_)/(*Ā*_*pos*_*− Ā*_*neg*_), where *A*_*cpd*_ represents the compound well’s absorbance and *Ā*_*pos*_ and *Ā*_*neg*_ reflect mean absorbances of positive and negative control wells. In contrast to prior sorbitol-based PSAC inhibitor screens, this screen used PhTMA-Cl with 200 μM furosemide to isolate residual transport; another important difference, 37°C incubation, was used because residual transport has a steep temperature dependence [24]. Although osmotic lysis in PhTMA-Cl requires net uptake of both PhTMA^+^ and Cl^-^, the higher permeability of Cl^-^ and electroneutrality considerations dictate that PhTMA^+^ uptake is rate limiting for osmotic lysis under these conditions.

The *Z’* statistic, calculated from mean and standard deviations of positive and negative control well absorbances, was used to evaluate assay reproducibility [27].

### Parasite growth inhibition assays and isobolograms

Efficacy of inhibitors against *in vitro* parasite growth was evaluated with the SYBR Green I assay for parasite nucleic acid as described [10]. Synchronous ring-stage cultures were seeded into 96-well microplates at 2% hematocrit and 0.5–1% parasitemia in either standard medium (RPMI 1640 medium with 25 mM HEPES, 0.5% NZ microbiological BSA, 50 mg/L hypoxanthine) or PGIM, which contains reduced concentrations of isoleucine (11.4 μM), glutamine (102 μM), and hypoxanthine (3.01 μM) but is otherwise identical [2]; inhibitors, where present, were added to indicated final concentrations before cultivation for 72 h. These cultures were then lysed (20 mM Tris, 10 mM EDTA, 0.016% saponin, 1.6% Triton X-100, pH 7.5) and incubated for 45 min with SYBR Green I nucleic acid stain at a 5000-fold dilution (Invitrogen, Waltham, MA). The mean fluorescence of replicate wells (excitation, 485 nm; emission, 528 nm) was used to quantify parasite DNA content and growth after normalization to in-plate controls with and without 20 μM chloroquine (100 and 0% growth inhibition, respectively).

Growth inhibition resulting from combinations of PSAC inhibitors was similarly evaluated using fixed ratios of the two compounds [28]. For each experiment, 9 fixed ratios of the two inhibitors (10:0, 9:1, …, 1:9, 0:10) were prepared based on the optimal concentrations of each in isolated growth inhibition dose responses. For each ratio, serial dilutions were used to generate dose responses and permit calculation of *IC*_*50*_ values by interpolation; in each experiment, matched dose responses were included for each compound in isolation. Isobolograms were generated using the corresponding concentrations of each inhibitor in the mixture by conventional methods [29], which indicate whether the two compounds interact to produce synergistic or antagonistic action against cell growth. For each inhibitor combination, synergistic action was quantified using the ∑FIC_50_ (sum of fractional inhibitor concentrations producing 50% block) [30]; values of this parameter below 0.7 indicate strong synergistic action.

## Results

### High throughput screen identifies novel inhibitors of residual transport through PSAC

**Fig 1:**
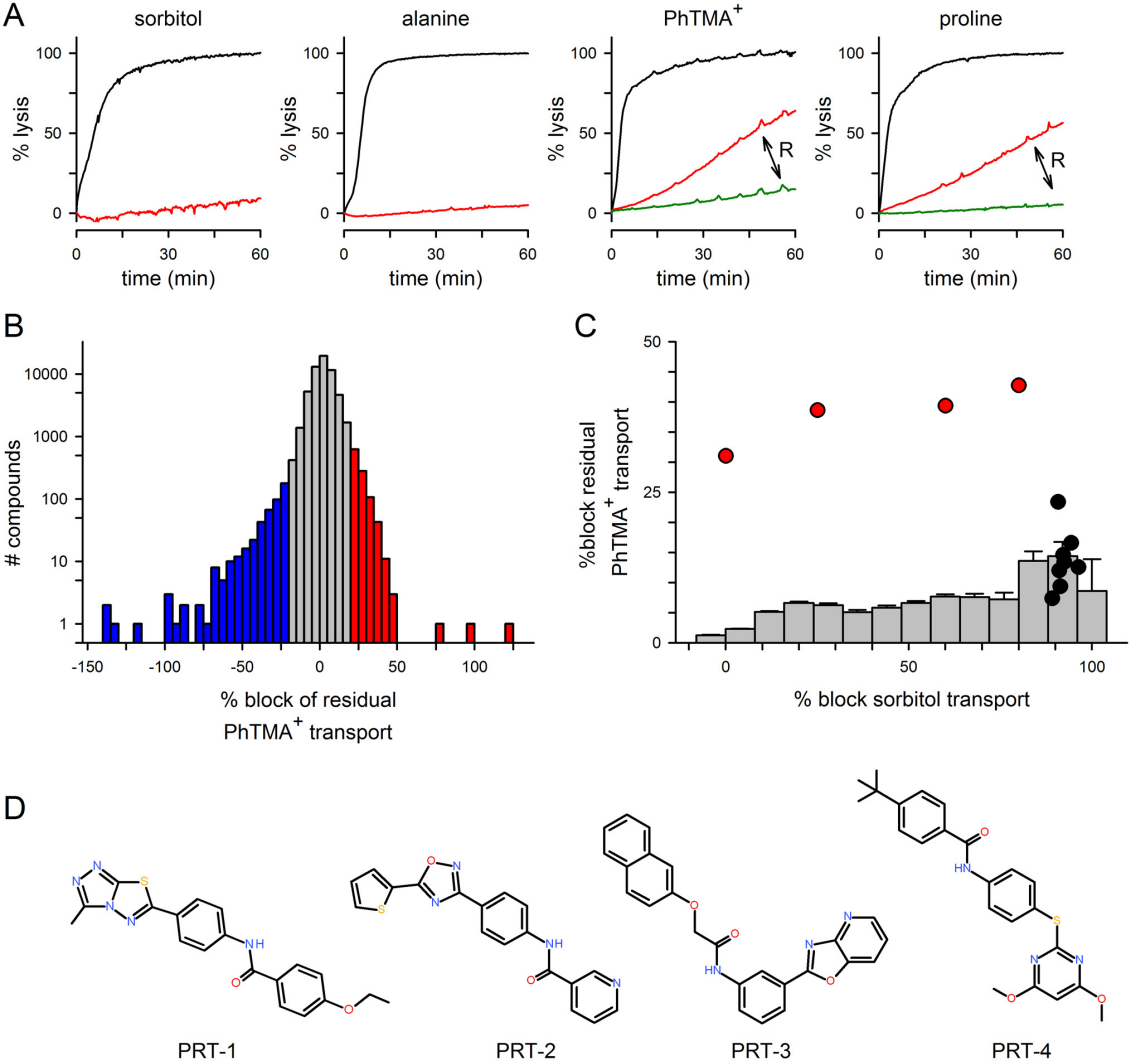
A cell-based high-throughput screen identifies novel residual transport inhibitors. (A) Kinetics of osmotic lysis in indicated solutes with 0, 200, or 2000 μM furosemide (black, red, and green traces where present in each panel, respectively). Notice that 200 μM furosemide abolishes lysis due to sorbitol or alanine uptake, but yields residual uptake of PhTMA^+^ and proline (indicated with arrow and “R”). (B) All points histogram of compound activities against osmotic lysis in PhTMA^+^ + 200 μM furosemide, as measured at 3 h and normalized to 0% block in DMSO-only wells and to 100% block in positive controls (HBS+1600 μM furosemide). Ordinate shows the number of compounds at each block level on a logarithmic scale, revealing that most compounds fell within 3*SD of negative control wells (grey bars). A small number of hits inhibited or accentuated lysis (red and blue bars, respectively). Two of the three compounds producing ≥ 50% block were unavailable for downstream experiments; the third did not reproduce in secondary studies. (C) Histogram comparing compound activities against primary and residual transport (*x* and *y* axes reflecting inhibition in parallel sorbitol and PhTMA^+^ + furosemide screens, respectively). Notice that mean ± SEM % activity against residual PhTMA^+^ transport gradually increases with activity in the sorbitol screen (grey histogram bars). Key hits reported previously in the sorbitol screen (compounds **1–8** from ref. [10]) exhibited weak activity in the PhTMA^+^ screen (black circles). Novel hits from the present screen are superimposed as red circles. (D) Structures and names of key hits from the PhTMA^+^ screen, with the prefix PRT referring to action against the PSAC residual transport.

Molecular studies have revealed that sorbitol, alanine, proline and the organic cation phenyl-trimethylammonium (PhTMA^+^) enter infected erythrocytes primarily via PSAC [11,12]. Despite evidence for a single shared channel, there are interesting discrepancies in transport block by available inhibitors. Fig 1A shows that 200 μM furosemide abolishes sorbitol and alanine uptake, but allows sufficient uptake of either PhTMA^+^ or proline to produce osmotic lysis of infected cells in kinetic measurements; a 10-fold higher concentration of furosemide abolishes this residual transport. PhTMA^+^, proline, and some other solutes are collectively referred to as *R*^+^ solutes (for residual-positive) because they consistently exhibit reduced efficacy block, despite use of PSAC inhibitors having diverse scaffolds and a broad range of potencies [10,24,25]; solutes such as sorbitol and alanine, which do not exhibit this effect, are known as *R*^-^ solutes. For each *R*^+^ solute, our analyses suggest that the residual transport component corresponds to 3 to 8% of the inhibitor-free uptake, as determined from an inverse relationship between permeability and the time to a threshold level of osmotic lysis [26]. To distinguish it from residual transport, we refer to the uptake blocked by 200 μM furosemide as “primary” transport; studies described below show that previously identified inhibitors, including furosemide, preferentially inhibit this primary component.

While osmotic lysis measurements require iso-osmolar concentrations of permeant solutes, tracer flux using physiological saline and low millimolar concentrations of permeant solutes confirm these pharmacological effects of *R*^-^ and *R*^+^solutes [24]; patch-clamp with high ionic strength solutions has also revealed reduced furosemide efficacy when PhTMA^+^ is present. These findings suggest two distinct mechanisms of permeation through PSAC and mitigate concerns about experimental conditions.

We therefore designed and executed a high-throughput screen for inhibitors of residual transport. We used a cell-based transmittance assay that tracks osmotic lysis in solutes having increased permeability after infection. As with previous screens [10,11], we used a 384-well microplate format and transmittance measurements to quantify osmotic lysis of cells in each well. While the prior screens used the *R*^-^ solute sorbitol and therefore identified potent and specific inhibitors of the primary transport component, the present screen sought inhibitors of the residual component and used the *R*^+^ solute PhTMA^+^. 200 μM furosemide was included in each compound well to isolate PhTMA^+^ residual transport; although addition of furosemide may adversely affect interactions of some compounds with the channel, this design was required to isolate the relatively modest contribution of the residual component. We screened 69,875 compounds from commercial libraries and available diversity-oriented synthesis collections at a high 10 μM concentration and read plates at multiple timepoints to maximize detection of hits. Matched positive and negative plates, run on each screening day, yielded a Z’ statistic of 0.60 ± 0.07, indicating a robust assay with an excellent signal-to-noise ratio [27].

Fig 1B tallies the all-points histogram of activities at the 3 h read, a timepoint that yields complete lysis of infected cells when residual transport is not blocked. 1.3% of the compounds exhibited activity outside of a threshold defined by 3*standard deviation of negative control wells. A large subset of hits yielded negative block (blue shading, Fig 1B), suggesting either detergent-like activity or agonistic activity on channel-mediated PhTMA^+^ uptake. ~0.8% of the compounds exhibited block above controls (red shading), suggesting inhibition of residual PhTMA^+^ transport in the presence of furosemide.

Because previous studies determined that residual transport can be inhibited by increased concentrations of primary component PSAC inhibitors, we recognized that some hits may reflect potent PSAC inhibitors without preferential action on the residual mechanism. To evaluate this possibility, we compared the results of the present screen to a prior *R*^-^ solute screen that used sorbitol without furosemide against the same collection of compounds [10]. Fig 1C shows that the mean normalized block in the PhTMA^+^ plus furosemide screen gradually increases with compound potency in the sorbitol screen (grey histogram bars), consistent with the observation that elevated concentrations of primary component inhibitors can inhibit residual PhTMA^+^ transport. Further analysis, nevertheless, implicated two distinct groups of inhibitors. Previously validated hits from the sorbitol screen are shown as black symbols superimposed on this histogram; as a group, these compounds averaged 92 ± 0.8% block in the sorbitol screen and only 13.7 ± 1.7% block in PhTMA+200 μM furosemide. A second group of hits, shown as red symbols, had greater activity in the PhTMA+200 μM furosemide screen, with normalized block values ≥ 31% in this screen and variable activities in the sorbitol screen. These compounds were chemically distinct from the scaffolds reported previously from sorbitol screens (Fig 1D).

### Secondary studies: improved activity against residual transport

**Fig 2:**
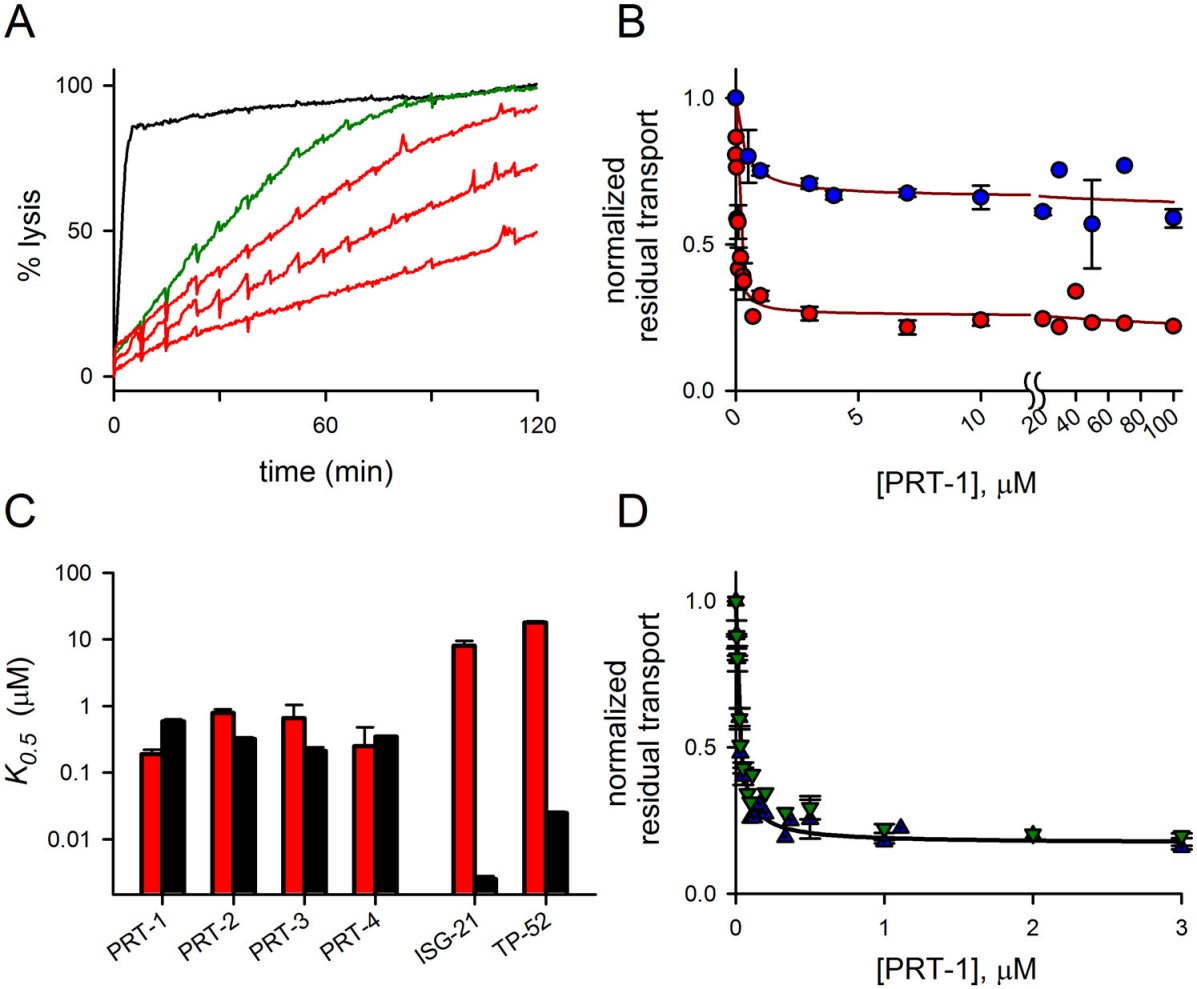
Hits act against residual transport and differ from known PSAC inhibitors. (A) Osmotic lysis kinetics in PhTMA lysis solution without (black trace) or with 200 μM furosemide (green or red traces). Addition of 0.1, 0.3, or 1 μM PRT-1 (top to bottom red traces, respectively) inhibits residual lysis. (B) Dose responses for inhibition of residual permeability (*P*) of PhTMA^+^ or proline (red and blue circles, respectively; mean ± S.E.M. of up to 10 trials each). (C) Inhibitor concentrations that block uptake by 50% (*K*_*0*.*5*_). Mean ± S.E.M. for inhibition of residual transport in PhTMA^+^ + 200 μM furosemide or primary transport in sorbitol are shown on a logarithmic scale as black and red bars, respectively. While ISG-21 and TP-52 have higher affinities against primary transport, PRT inhibitors have comparable or greater activity against residual transport. (D) Dose responses for PRT-1 inhibition of residual *P* in PhTMA^+^ plus either 100 nM ISG-21 or 2 μM TP-52 (blue and green triangles, respectively; mean ± S.E.M. of up to 3 trials each). Solid lines in panels (B) and (D) represent the best fits to the sum of two Langmuir isotherms [11].

We next used kinetic measurements with the new inhibitors to examine potency and mechanism of action. The hit compound PRT-1 potently inhibited residual transport in secondary studies (Fig 2A and 2B); dose response studies revealed that this hit blocked residual PhTMA^+^ uptake with a *K*_*0*.*5*_ of 190 ± 3 nM when examined in combination with 200 μM furosemide. Because this affinity was higher than that for inhibition of the primary channel route (*K*_*0*.*5*_ of 590 ± 43 nM against sorbitol uptake; red vs. black bars, Fig 2C; *P <* 10^−4^, Student’s *t* test), PRT-1 preferentially inhibits residual transport. Each of the hits described in Fig 1D exhibited submicromolar affinity for residual transport, matching or exceeding their activities against sorbitol uptake (Fig 2C). In contrast, ISG-21 and TP-52, potent PSAC inhibitors from separate scaffolds identified in previous primary component screens [10,11], exhibited a strong preferential inhibition of sorbitol uptake and were up to 3000-fold less effective against residual PhTMA^+^ transport (Fig 2C; *P <* 10^−4^, comparisons of *K*_*0*.*5*_ values for each inhibitor, Student’s *t* test). The differing activities of inhibitors against uptake of these solutes validates our cell-based screening and is consistent with the model of two distinct transport mechanisms through PSAC.

This model was further tested by evaluating PRT-1 block in the presence of other primary component inhibitors: if it blocks residual transport only in combination with furosemide, this would suggest interactions limited to these two inhibitors. We therefore identified ISG-21 and TP-52 concentrations that abolish sorbitol uptake but yield significant PhTMA^+^ uptake and osmotic lysis (S1 Fig). PRT-1 dose responses using PhTMA-Cl and threshold levels of these primary component inhibitors revealed potent block of residual transport (Fig 2D), excluding an effect specific to combination with furosemide. The PRT-1 *K*_*0*.*5*_ values, 44 and 56 nM when evaluated with ISG-21 or TP-52, indicated significantly improved potency (*P* < 0.01 for comparisons to combination with furosemide). It is possible that these more potent primary component inhibitors promote accessibility of PRT-1 to its site on the channel; molecular and structural studies to explore this improvement are warranted.

We next examined PRT-1 efficacy against the residual transport of proline (Fig 1A), an *R*^+^ solute structurally unrelated to PhTMA^+^. Although PRT-1 clearly reduced proline transport further when added to 200 μM furosemide (blue circles, Fig 2B), the maximal inhibition reached was less than that observed with PhTMA^+^ transport (*P* < 10^−4^ for comparisons at 10 μM PRT-1, *n* = 5–7 trials each). This unexpected difference reveals a further complexity in how the channel may recognize and transport individual *R*^+^ solutes.

PSAC activity is conserved in all studied *Plasmodium* species, with single channel conductance and gating properties almost indistinguishable between phylogenetically distant pathogens [18]. Using rhesus monkey erythrocytes infected with *P*. *knowlesi*, we found that both PhTMA^+^ and proline exhibit residual transport with 200 μM furosemide while sorbitol and alanine uptake are abolished (panel A, S2 Fig). This finding implicates conservation of the two mechanisms of transport through PSAC, despite the substantial evolutionary distance between *P*. *falciparum* and *P*. *knowlesi* [31]. Importantly, we also found that PRT-1 and PRT-3 both inhibit residual PhTMA^+^ transport via the *P*. *knowlesi* channel (panel B, S2 Fig), adding to the high-level conservation of this phenotype in malaria parasites.

### A PRT-1 derivative with improved potency

**Fig 3:**
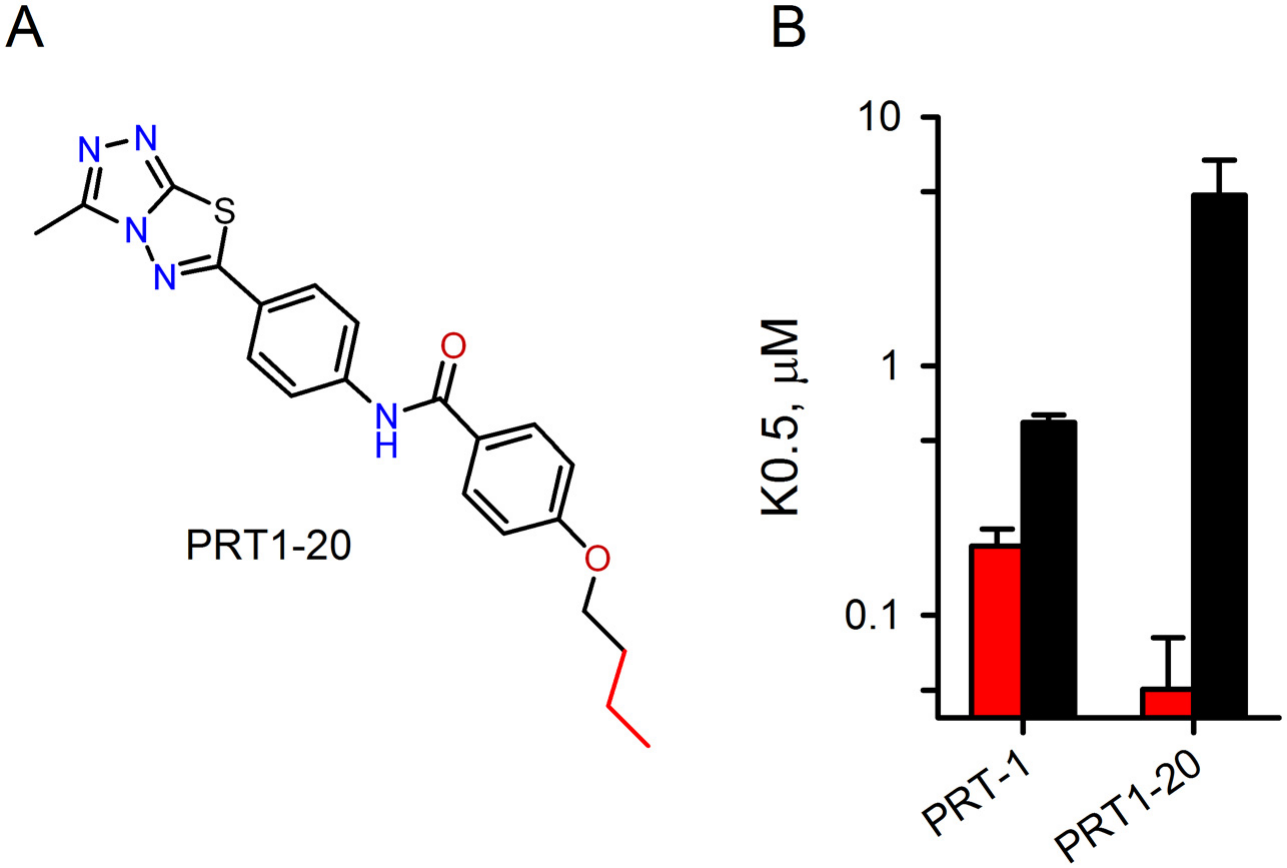
A PRT-1 derivative with improved potency and specificity. (A) Structure of PRT1-20. A longer alkoxy side chain distinguishes this compound from PRT-1 (red highlight). (B) Mean ± S.E.M. inhibitor *K*_*0*.*5*_ values in PhTMA^+^ + 200 μM furosemide (red bars) and sorbitol (black). When compared to PRT-1, PRT1-20 has improved greater potency against residual transport and reduced activity against the primary mechanism.

To explore structure-activity relationship for residual transport inhibition, we next carried out a secondary screen of available PRT-1 derivatives and identified a 4-butoxy analog with improved specificity of action (Fig 3A). This compound, PRT1-20, exhibited both improved activity against residual PhTMA^+^ transport and significantly reduced action against the primary transport component (Fig 3B, *P* = 0.02 for comparisons between PRT-1 and PRT1-20 in dose response studies using either PhTMA^+^ with 200 μM furosemide or sorbitol without furosemide). The combination of improved activity against residual PhTMA^+^ uptake and reduced potency against sorbitol uptake further supports the model of two separate mechanisms of transport through the channel.

### Synergistic parasite killing with combinations of inhibitors from separate classes

**Fig 4:**
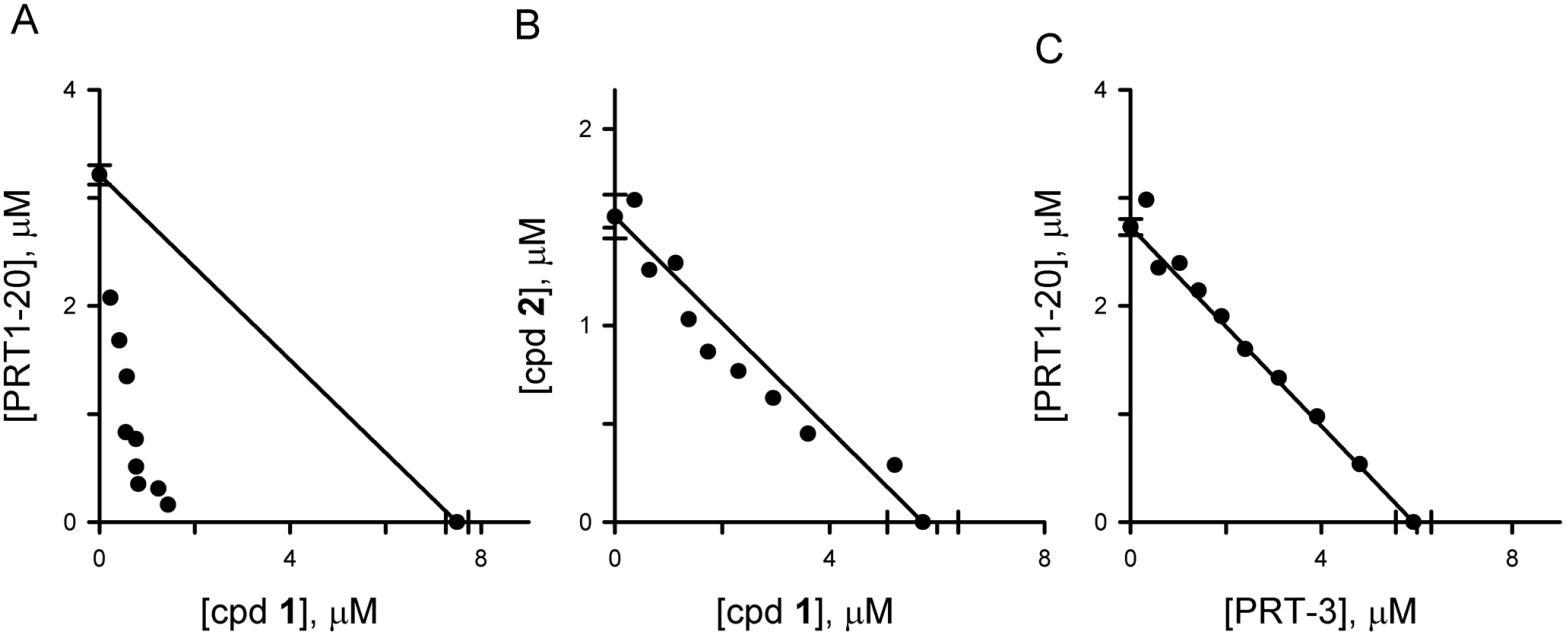
Synergistic killing by combinations of primary and residual inhibitors, but not by combinations from one class only. (A) Isobologram showing effect of fixed ratio combinations of the primary component inhibitor cpd **1** and the residual transport inhibitor PRT1-20. Each symbol represented the *IC*_*50*_ for a fixed ratio of the two inhibitors, determined from a full dose response experiment with replicates. Error bars, shown for single compound *IC*_*50*_ values (symbols on the *x* and *y* axes), represent S.E.M. values. Solid line connecting these intercept values is the expected profile for additive parasite killing. Strong synergistic killing was found for this drug combination as the symbols are markedly below the additive line. (B) Isobologram for two primary component inhibitors, showing additive interaction. (C) Isobologram for two residual component inhibitors, showing additive interaction.

Inhibitor-mediated PSAC block interferes with parasite development, as evidenced by genetic mapping of *in vitro* growth inhibition to the channel-associated *clag3* locus and by correlations between inhibitor affinity and parasite killing [2,10]. Improved killing by PSAC inhibitors upon nutrient restriction in parasite culture medium has implicated an essential role of the channel in parasite nutrient uptake. We therefore asked whether the new residual inhibitors also interfere with parasite growth. Because residual transport may represent a distinct route for nutrient uptake, we hypothesized that simultaneous inhibition of both channel mechanisms may produce a greater effect on parasite growth. To examine this possibility, we combined cpd **1**, a previously identified primary transport component inhibitor (S3 Fig)[10], with PRT1-20 and evaluated parasite growth inhibition. The fixed ratio approach was used to estimate *IC*_*50*_ values in dose response experiments; isobologram analysis is shown in Fig 4A. Both cpd **1** and PRT1-20 inhibited parasite growth at low micromolar concentrations when applied individually in standard RPMI-1640 based media (*x-* and *y*-intercepts, respectively). Combinations of the two compounds produce strongly synergistic growth inhibition, as indicated by the *IC*_*50*_ values below the additive interaction line (solid diagonal line, Fig 4A). For example, 50% parasite growth inhibition was achieved with a combination of 0.82 μM cpd **1** and 0.35 μM PRT1-20, concentrations that are approximately 10% of each compound’s *IC*_*50*_ value in single drug growth experiments. The mean ∑FIC_50_ statistic for this drug combination, 0.38 ± 0.06, corresponds to strong synergy (*P* < 10^−4^ for the no-interaction hypothesis, *t* test).

We next examined multiple combinations of PSAC inhibitors identified from prior primary component screens and the present residual component screen (Table 1). These studies revealed that most inhibitor combinations that target both transport components were strongly synergistic with mean ∑FIC_50_ values between 0.29 and 0.74 (orange shading in Table 1), matching or exceeding those measured with highly synergistic drug combinations used for malaria therapy [30]. Lower degrees of synergism for some combinations may reflect effects of relative inhibitor affinities, competition for overlapping binding sites on the channel, off-target effects of some compounds, or variable inhibitor stability under culture conditions.

**Table 1:** *In vitro* interactions between primary and residual PSAC inhibitors. Combination growth inhibition experiments, tallied as mean ± S.E.M. sum of FIC values for 50% growth inhibition (∑FIC_50_). Combinations are grouped under headings to indicate experiments performed with a primary and a residual inhibitor, two primary inhibitors, or two residual inhibitors.

Inhibitor 1	Inhibitor 2	∑FIC_50_
Primary and residual inhibitor combinations		
cpd **1**	PRT-1	0.40 ± 0.03
cpd **1**	PRT1-20	0.38 ± 0.06
cpd **1**	PRT-2	0.90 ± 0.07
cpd **1**	PRT-3	0.41 ± 0.03
cpd **2**	PRT-1	0.54 ± 0.05
cpd **2a**	PRT-1	0.57 ± 0.05
cpd **2a**	PRT-2	0.94 ± 0.02
cpd **2a**	PRT-3	0.57 ± 0.02
CD012	PRT-1	0.36 ± 0.04
THM-191	PRT-1	0.74 ± 0.07
TP-52	PRT-1	0.57 ± 0.07
TP-52	PRT1-20	0.29 ± 0.03
ISG-21	PRT-1	0.51 ± 0.10
Two primary inhibitors		
cpd **1**	cpd **2**	0.97 ± 0.03
cpd **1**	cpd **2a**	0.82 ± 0.03
cpd **2**	THM-191	1.32 ± 0.07
cpd **2a**	CD012	0.93 ± 0.02
Two residual inhibitors		
PRT-1	PRT-3	1.02 ± 0.08
PRT1-20	PRT-2	0.87 ± 0.04
PRT1-20	PRT-3	1.02 ± 0.02

In contrast, combinations that target only one of the two transport components were consistently additive (Fig 4B and 4C; Table 1, blue and yellow shading for combinations of only primary and only residual inhibitors, respectively). The reproducible pattern of synergistic killing with combinations that include both a primary and a residual transport inhibitor confirms that the present screen yields inhibitors with properties distinct from known PSAC inhibitors and further supports the model of distinct transport mechanisms through a shared channel. These findings also indicate that these compounds kill parasites through action on PSAC rather than through effects on unrelated parasite targets, which could not produce the observed pattern of synergistic killing. Finally, they suggest that the parasite uses the residual transport route to acquire certain nutritive solutes under *in vitro* culture conditions.

### Effects of extracellular nutrient availability

**Fig 5:**
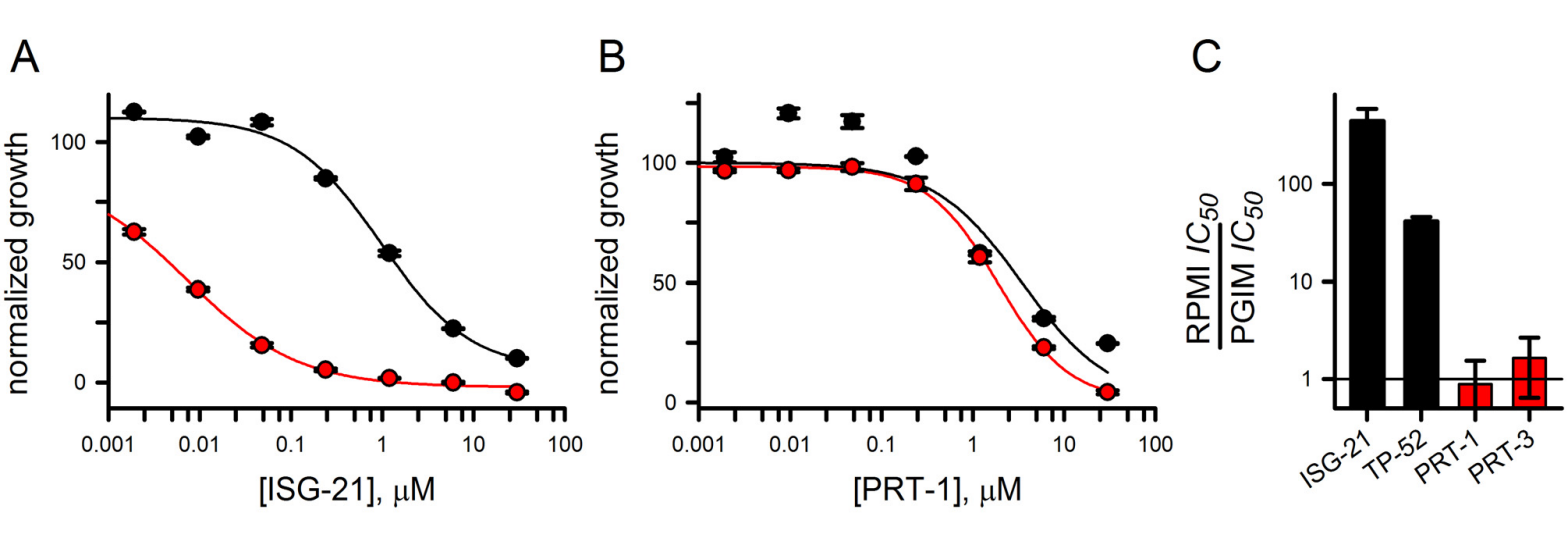
Effects of external nutrient levels on inhibitor efficacy against parasite growth. (A, B) Dose responses for growth inhibition by ISG-21 and PRT-1 in standard medium and PGIM (black and red symbols, respectively). While ISG-21 has significantly improved activity in PGIM, the efficacy of PRT-1 is unchanged. Solid lines represent best fits to a logistic decay with a Hill coefficient. (C) Ratio of *IC*_*50*_ values for parasite killing in standard RPMI 1640-based medium to PGIM for indicated inhibitors. Bars represent mean ± S.E.M. of replicates from up to 7 independent trials.

We next examined whether nutrient availability affects the activity of residual transport inhibitors. Inhibitors of the primary transport component are more effective against *in vitro* parasite growth when tested using PGIM, a modified medium that follows the commercial RPMI-1640 formulation but has reduced levels of isoleucine, glutamine, and hypoxanthine, three important nutrients with uptake primarily via PSAC [2,32–34]. For those inhibitors, a 10- to 800-fold improvement in *IC*_*50*_ values was observed (Fig 5A and 5C, tallied from the present and prior studies [2]). In contrast, the residual inhibitors PRT-1 and PRT-3 did not show measurable changes in efficacy in PGIM (Fig 5B and 5C). This difference further distinguishes the two broad classes of PSAC inhibitors. Failure to show improvement upon reduction of extracellular nutrients presumably reflects the relatively modest contribution of the residual component to overall channel-mediated uptake.

**Fig 6:**
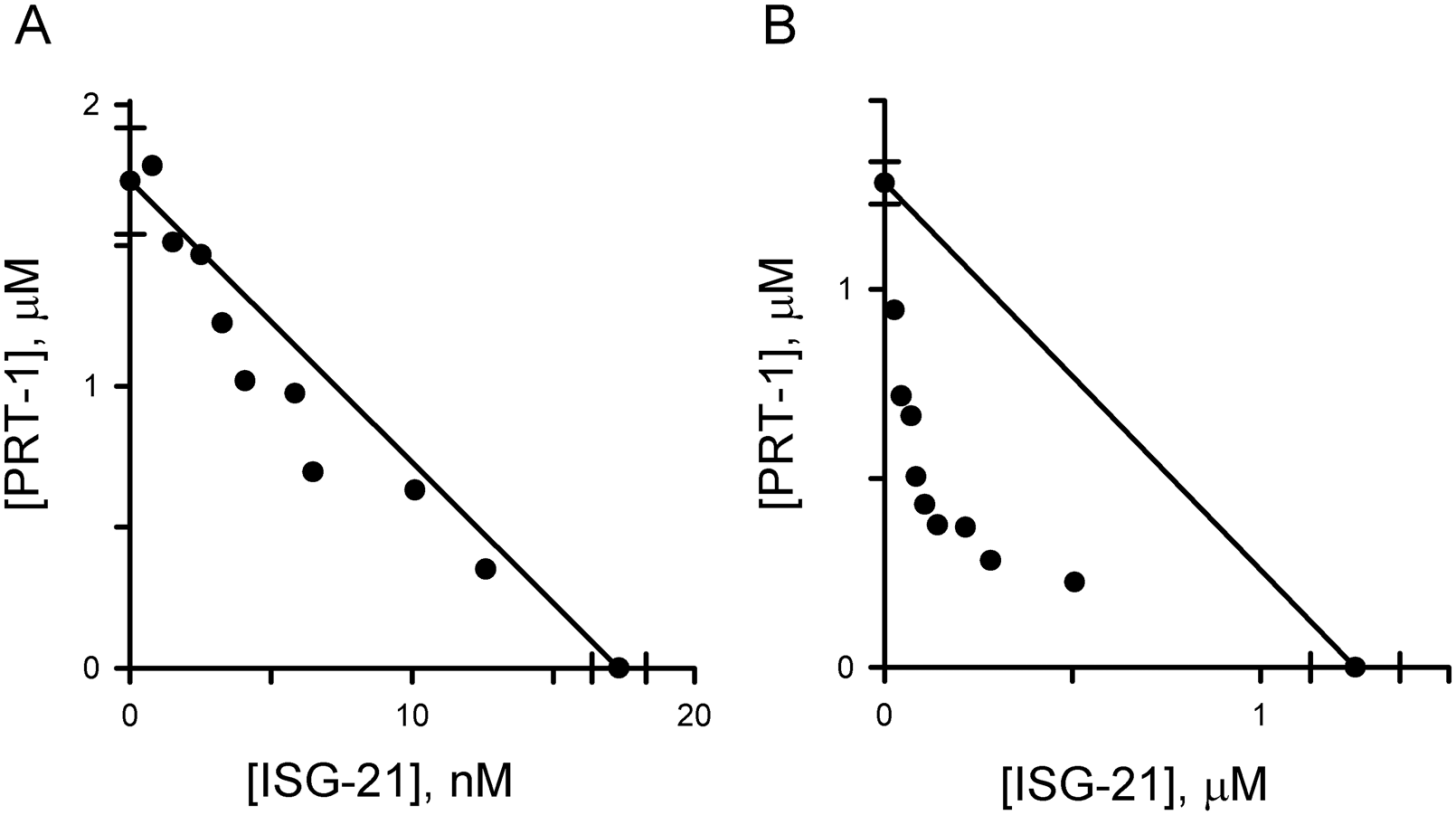
Effect of external nutrient levels on synergistic killing. (A) Isobologram for ISG-21 and PRT-1 interactions in PGIM. Note that synergistic action is lost in this medium. (B) Isobologram for an identical growth experiments after increasing the culture medium isoleucine concentration to 34 μM, showing restored synergy. The ISG-21 *IC*_*50*_ values in (A) and (B)—17.3 ± 1 nM and 1.25 ± 0.1 μM, *x* axis intercepts—differ markedly because extracellular nutrient levels affect killing by primary component blockers.

To further explore the effect of nutrient availability, we evaluated inhibitor combinations and found, remarkably, that the marked synergy between primary and residual component inhibitors in standard media was abolished when evaluated using PGIM. The mean ∑FIC_50_ values for PRT-1 in combination with either ISG-21 or cpd **2** in PGIM, 0.92 ± 0.03 and 0.94 ± 0.03, confirmed that neither produced synergy upon nutrient restriction (Fig 6A, isobologram for interactions with ISG-21; data for interactions with cpd **2** not shown). A modest increase in the extracellular concentration of isoleucine, an essential amino acid taken up via PSAC as an *R*^+^ solute [25,35], from its PGIM value of 11 μM to 34 μM largely restored synergy (Fig 6B, ∑FIC_50_ = 0.52 ± 0.04), revealing a specific nutrient that uses the residual mechanism under physiological conditions. Thus, *in vitro* parasite survival and propagation is dependent on both external nutrient availability and transport through PSAC via one of two mechanisms.

## Discussion

The model of two distinct transport mechanisms through PSAC motivated our present high-throughput screen and secondary studies. Key findings from these studies include: 1) the new screen using PhTMA^+^ with 200 μM furosemide yielded hits distinct from those found in prior screens using sorbitol; 2) secondary studies with these hits revealed greater activity against residual PhTMA^+^ transport, with similar behavior against channels from the divergent *P*. *knowlesi* parasite; 3) a small secondary screen identified PRT1-20, a compound with improved affinity against residual transport and a concomitant loss of activity against the primary transport mechanism; 4) *in vitro* growth inhibition studies revealed that the new inhibitors act synergistically with prior channel blockers, whereas combinations from a single inhibitor class produce only additive killing; 5) differing effects of nutrient restriction on growth inhibitory efficacy further distinguished these residual transport inhibitors from prior inhibitors.

What do these findings reveal about permeation through PSAC and differing effects of *R*^+^ and *R*^-^ solutes on inhibitor action? Although macroscopic transport measurements as used here cannot conclusively define the structural basis of permeation, the segregation of inhibitors into two unambiguous groups based on distinct chemical screens is consistent with a model that proposes two mechanisms of solute transport through the channel. These mechanisms may correspond to physically separate routes within a single channel complex, or they may instead reflect distinct binding sites for subsets of solutes along a common transit pathway. Physically separate routes have been described in several other channels [36,37], with some cases involving an aqueous pore and a carrier with alternating access [38]. With two-pore channels, it is tempting to predict that some inhibitors should have activity against one route and have no measurable action against the other pathway, but such route-specific inhibitors may not exist if the pore(s) undergo conformational changes with solute permeation. Nevertheless, because none of the primary or residual transport inhibitors found in various high-throughput screens have met this stringent criterion yet, we tend to favor a common transit pathway with distinct recognition sites for the two types of solutes. The observation that PRT-1 has differing efficacies against the residual transport of proline and PhTMA^+^ even though both are *R*^+^ solutes also argues against simple models with two physically separate routes (Fig 2B). The precise structural basis of residual transport and the differences in activities of the two types of PSAC inhibitors will require advances in available experimental approaches; site-directed mutagenesis of PSAC-associated parasite genes, heterologous expression of functional parasite channels, and structure determination may all be necessary.

Why does this parasite channel have two distinct mechanisms to transport solutes across the host membrane? Conservation of differences in *R*^-^ and *R*^+^ solute transport and the activities of residual inhibitors in the divergent *P*. *knowlesi* parasite suggests that the residual phenomenon is central to PSAC function (S2 Fig). We speculate that it may be a byproduct of essential channel design. Most importantly, PSAC has a remarkable selectivity profile with broad permeability to organic and inorganic solutes, but it excludes the small Na^+^ ion. This unprecedented combination presumably evolved to meet the parasite’s nutrient demands and to avoid osmotic lysis of infected cells in plasma, where Na^+^ is the predominant osmolyte. Although Na^+^ exclusion is partially achieved through electrostatic repulsion of cations at the pore mouth [39], the pore’s ability to distinguish structurally similar solutes and transport them by two separate mechanisms as implicated by our findings likely contributes to PSAC’s selectivity profile.

Despite these mechanistic uncertainties, the novel residual transport inhibitors identified here do provide insights into parasite nutrient acquisition and the role played by this unusual ion channel. The robust synergism with primary component PSAC inhibitors suggests that the residual transport mechanism is used by parasites under *in vitro* culture conditions. The loss of synergistic action upon nutrient restriction and significant restoration with addition of isoleucine implicate a role of both transport components in nutrient uptake. Synergistic action is especially valuable with antimalarial therapies, because combination drug therapies are now universally used and synergism may reduce the risk of acquired drug resistance [40]. These findings should further stimulate drug discovery against this attractive and essential target on the host erythrocyte membrane.

An important concern in antimalarial drug discovery is the possible action of screening lead compounds on unrelated parasite targets. Such “off-target effects” plague both molecular studies of parasite activities and antimalarial drug development efforts [41]. For example, phloridzin, a nonspecific inhibitor of PSAC and various other transporters [42], sterilizes parasite cultures and was initially thought to act primarily on PSAC because its *in vitro* growth *IC*_*50*_ and transport inhibition *K*_*0*.*5*_ values were nearly identical [43]. Subsequent selection of phloridzin-resistant parasites and functional studies however revealed that other parasite activities are the main targets of this agent [44]. Further studies of the new inhibitors, as well as of existing PSAC inhibitors, are required to exclude such off-target effects and allow advancement into the antimalarial drug development pipeline. However, the pattern of synergistic killing observed for combinations of residual and primary component inhibitors but additive-only killing for inhibitor pairs from a single group strongly suggests direct action on PSAC; this pattern cannot be readily explained by off-target effects. Loss of this synergism upon isoleucine restriction is also consistent with our observation that this essential nutrient exhibits *R*^+^ transport behavior in both osmotic lysis experiments and tracer flux using more physiological buffers [24,45].

A better understanding of both the structural basis of permeation and transport inhibition by the two classes of blockers will be critical for development of novel therapies targeting parasite nutrient uptake.

## Supporting Information

S1 Fig ISG-21 and TP-52 are potent primary component inhibitors. (A) Osmotic lysis kinetics in indicated solutes without and with 100 nM ISG-21 (black and red traces, respectively). (B) Lysis kinetics without and with 2 μM TP-52 (black and red traces, respectively). The selected concentrations of these inhibitors abolish sorbitol uptake, but yield residual uptake of PhTMA^+^.
(PDF)

S2 Fig Similar transport of solutes and residual inhibitor action in rhesus erythrocytes infected with *P*. *knowlesi*. (A) Osmotic lysis kinetics for *P*. *knowlesi*-induced channels in indicated solutes without and with 200 μM furosemide (black and red traces, respectively). (B) Inhibition of residual transport in *P*. *knowlesi*-infected cells by 3 μM PRT-1 or 3 μM PRT-3 (blue and green traces, respectively; other traces as in panel A).
(PDF)

S3 Fig Structures of primary component inhibitors used in these studies. (PDF)
