# Supplementary material for: Synergistic Malaria Parasite Killing by Two Types of Plasmodial Surface Anion Channel Inhibitors
Source: PLoS One. 2016 Feb 11;11(2):e0149214. doi: 10.1371/journal.pone.0149214 (PMC4750852; doi:10.1371/journal.pone.0149214)
Supplement: S1 Fig — (A) Osmotic lysis kinetics in indicated solutes without and with 100 nM ISG-21 (black and red traces, respectively). (B) Lysis kinetics without and with 2 μM TP-52 (black and red traces, respectively). The selected concentrations of these inhibitors abolish sorbitol uptake, but yield residual uptake of PhTMA+. (PDF) [file pone.0149214.s001.pdf]

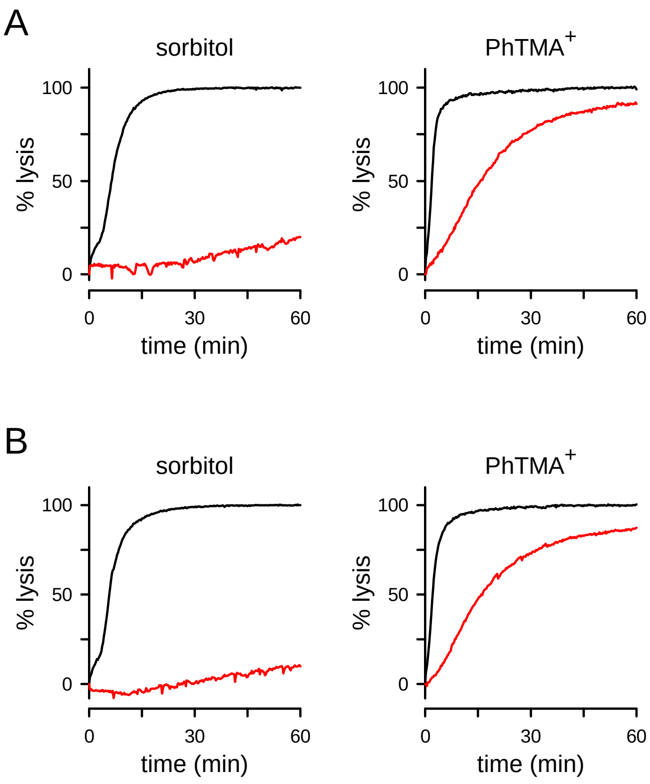

S1 Fig. ISG-21 and TP-52 are potent primary component inhibitors. (A) Osmotic lysis kinetics in indicated solutes without and with 100 nM ISG-21 (black and red traces, respectively). (B) Lysis kinetics without and with 2 μM TP-52 (black and red traces, respectively). The selected concentrations of these inhibitors abolish sorbitol uptake, but yield residual uptake of $PhTMA^+$.
