# Supplementary material for: Synergistic Malaria Parasite Killing by Two Types of Plasmodial Surface Anion Channel Inhibitors
Source: PLoS One. 2016 Feb 11;11(2):e0149214. doi: 10.1371/journal.pone.0149214 (PMC4750852; doi:10.1371/journal.pone.0149214)
Supplement: S2 Fig — (A) Osmotic lysis kinetics for P. knowlesi-induced channels in indicated solutes without and with 200 μM furosemide (black and red traces, respectively). (B) Inhibition of residual transport in P. knowlesi-infected cells by 3 μM PRT-1 or 3 μM PRT-3 (blue and green traces, respectively; other traces as in panel A). (PDF) [file pone.0149214.s002.pdf]

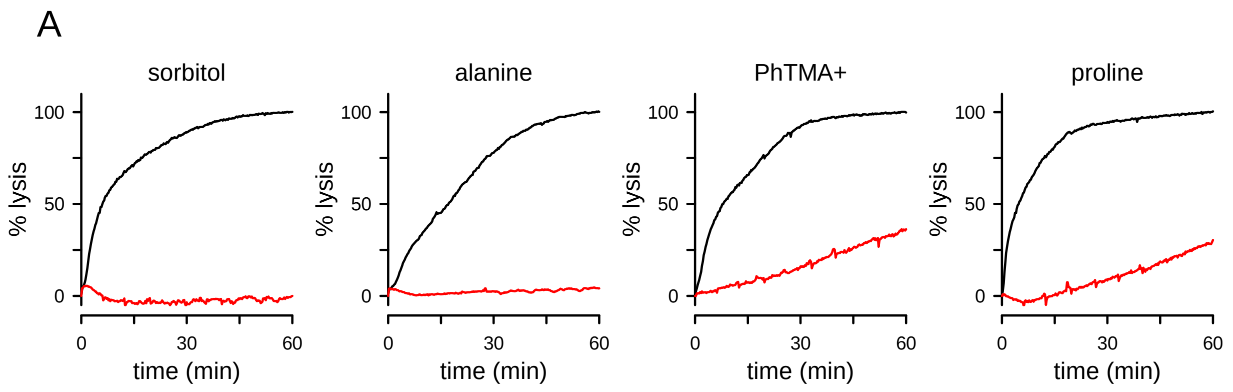

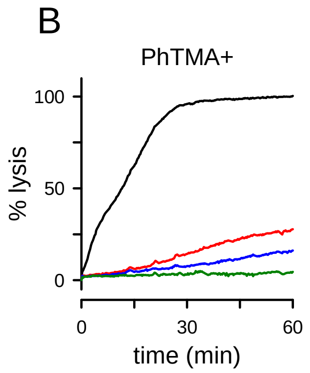

S2 Fig. Similar transport of solutes and residual inhibitor action in rhesus erythrocytes infected with *P. knowlesi*. (A) Osmotic lysis kinetics for *P. knowlesi*-induced channels in indicated solutes without and with 200 µM furosemide (black and red traces, respectively). (B) Inhibition of the residual transport in *P. knowlesi*-infected cells by 3 µM PRT-1 or 3 µM PRT-3 (blue and green traces, respectively; other traces as in panel A).
