# Supplementary figures and images for: Synergistic Malaria Parasite Killing by Two Types of Plasmodial Surface Anion Channel Inhibitors
Source: PLoS One. 2016 Feb 11;11(2):e0149214. doi: 10.1371/journal.pone.0149214 (PMC4750852; doi:10.1371/journal.pone.0149214)

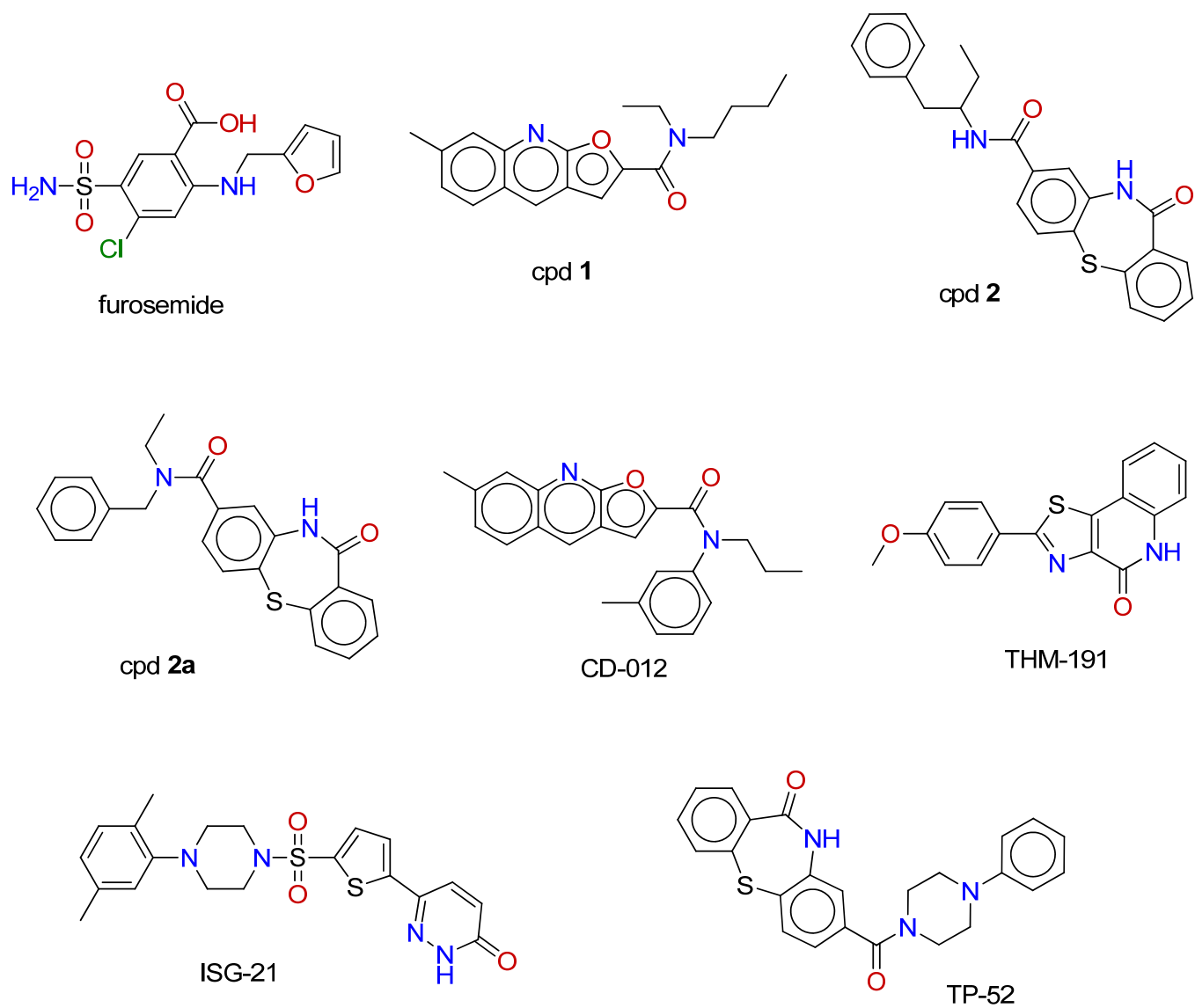

**S3 Fig. Structures of primary component inhibitors used in these studies.**

Supplement: S3 Fig — (PDF) [file pone.0149214.s003.pdf]
